# Supplementary material for: Monitoring Myelin Lipid Composition and the Structure of Myelinated Fibers Reveals a Maturation Delay in CMT1A
Source: Int J Mol Sci. 2024 Oct 19;25(20):11244. doi: 10.3390/ijms252011244 (PMC11508568; doi:10.3390/ijms252011244)
Supplement: Supplementary file 1 [file ijms-25-11244-s001.zip › Supplementary Figures and Methods.pdf]

Monitoring Myelin Lipid Composition and the Structure of Myelinated Fibers Reveals a Maturation Delay in CMT1A

Giovanna Capodivento [1,†], Mattia Camera [2,†], Nara Liessi [3], Anna Trada [2], Doriana Debellis [4], Angelo Schenone [1,2], Andrea Armirotti [3], Davide Visigalli [1,2,‡] and Lucilla Nobbio [1,*,‡]

1 IRCCS Ospedale Policlinico San Martino, Largo Rosanna Benzi 10, 16132, Genova, Italy
2 Department of Neurosciences, Rehabilitation, Ophthalmology, Genetics, and Maternal and Children's Sciences (DINOGMI), University of Genoa, 16126, Genova, Italy
3 Analytical Chemistry Facility, Istituto Italiano di Tecnologia, Via Morego 30, 16163, Genova, Italy
4 Electron Microscopy Facility, IIT, Via Morego 30, 16163, Genoa, Italy
† These authors contributed equally.
‡ These authors also contributed equally.
* Correspondence: lucilla.nobbio@hsanmartino.it

Supplementary Figures

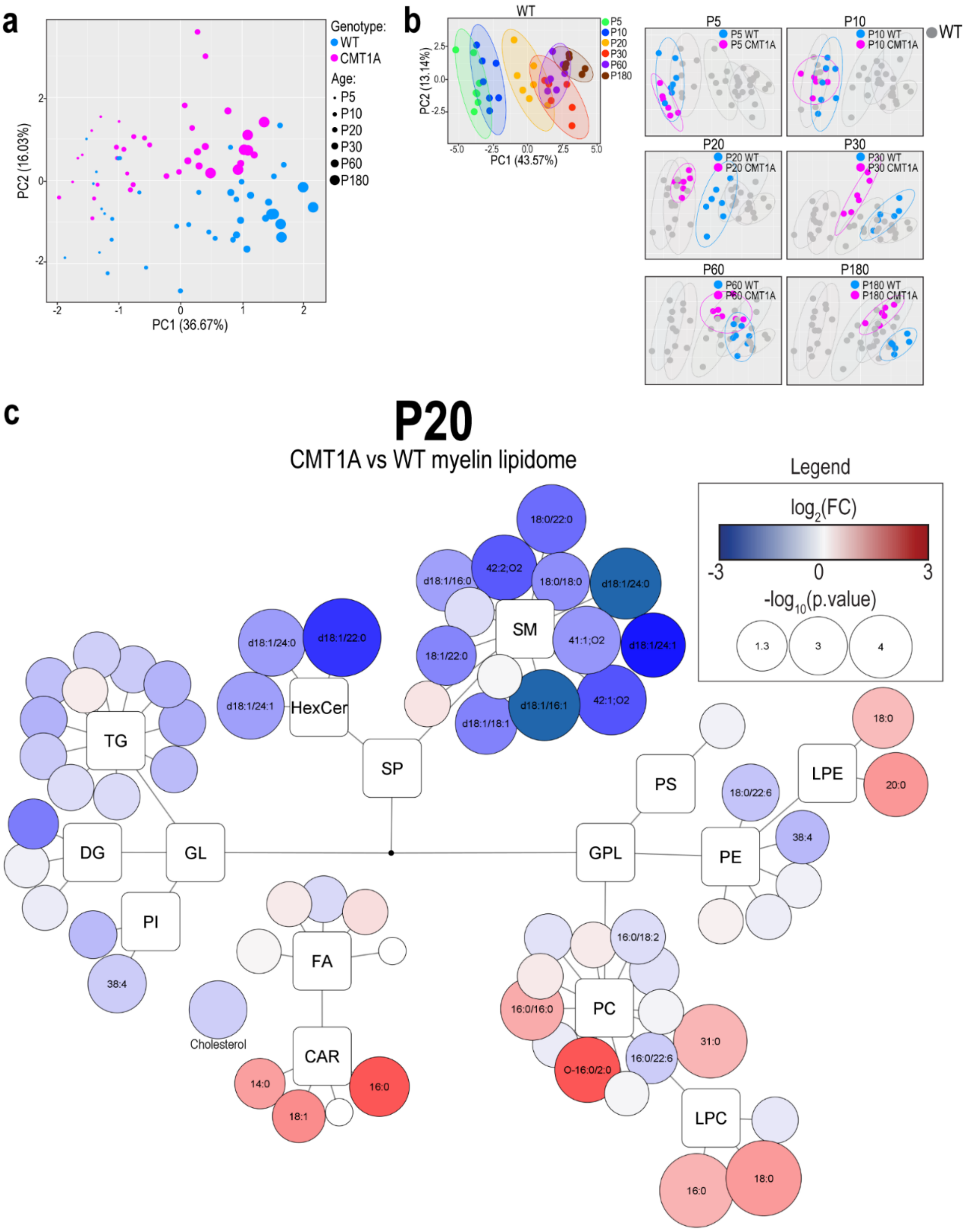

Supplementary Figure S1. Lipidomics Data. (**a**) PCA plot of lipidomic data from WT and CMT1A purified peripheral myelin extracted from the sciatic nerve of P5, P10, P20, P30, P60, and P180 rats. n = at least 6 samples for each group; for each sample, several nerves from different rats were pulled together to obtain enough myelin. (**b**) PCA plots showing CMT1A samples from each time point together with WT samples, to highlight the maturation delay experienced by CMT1A myelin. (**c**) Simplified network visualization of lipid metabolism showing alterations in myelin lipid profile of P20 CMT1A rats, as compared to WT. Each dot represents a lipid species, dot size expresses the significance according to *p* value, while the color intensity defines the degree of up (red) and downregulation (blue) according to the fold change (as CMT1A vs WT). The lipids showing a statistically significant difference are annotated. WT and CMT1A samples, n = 7; for each sample, sciatic nerves from 5 different animals were pulled together to obtain enough material for the analysis. *p* values were calculated using a two-tailed unpaired Student's t-test.

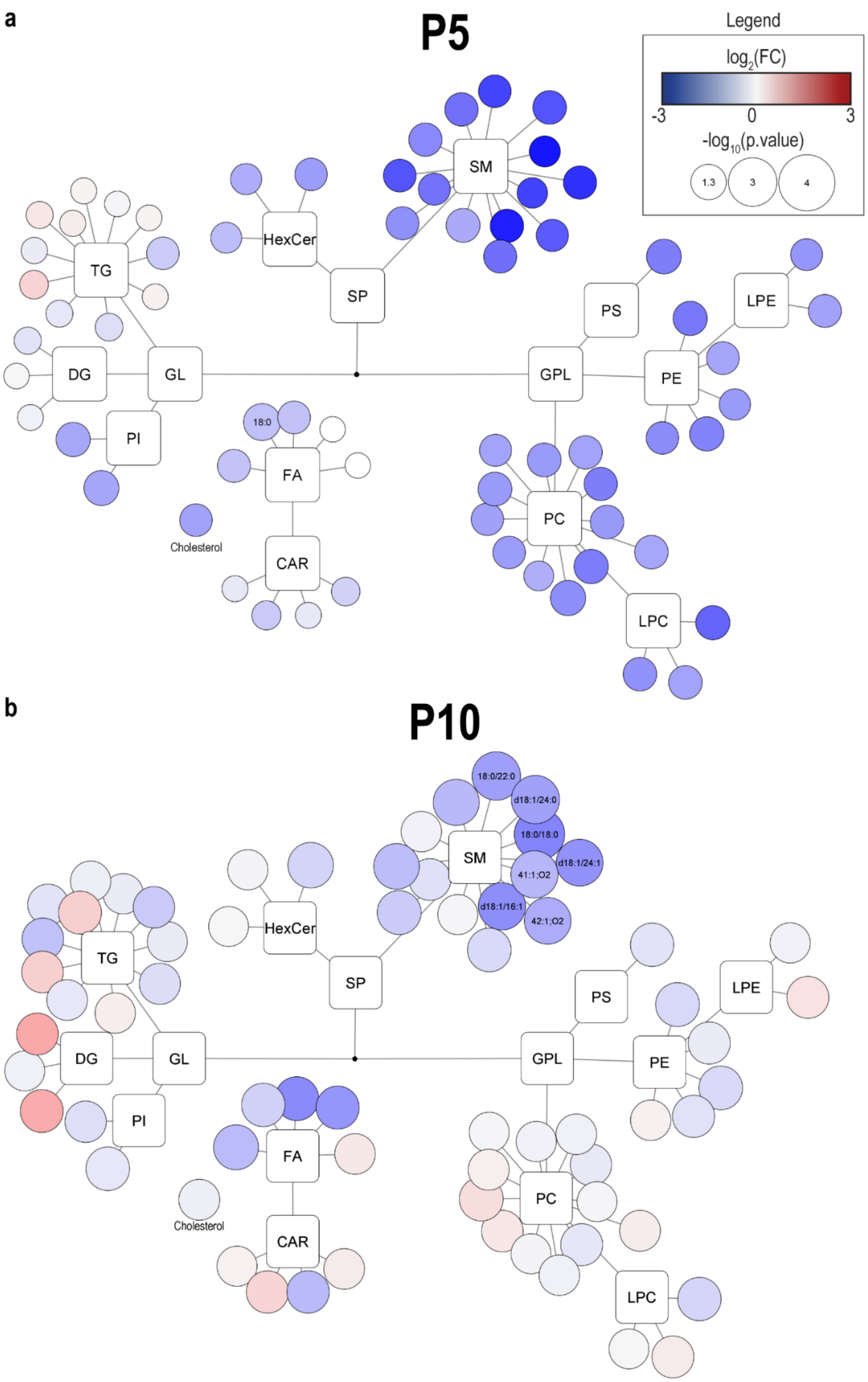

Supplementary Figure S2. Lipidomics Data; P5 and P10. Simplified network visualization of lipid metabolism showing alterations in myelin lipid profile of P5 (**a**) and P10 (**b**) CMT1A rats, as compared to WT. Each dot represents a lipid species, dot size expresses the significance according to *p* value, while the color intensity defines the degree of up (red) and downregulation (blue) according to the fold change (as CMT1A vs WT). The lipids showing a statistically significant difference are annotated. WT and CMT1A samples, n = 7; for each sample, sciatic nerves from 5 different animals were pulled together to obtain enough material for the analysis. *p* values were calculated using a two-tailed unpaired Student's t-test.

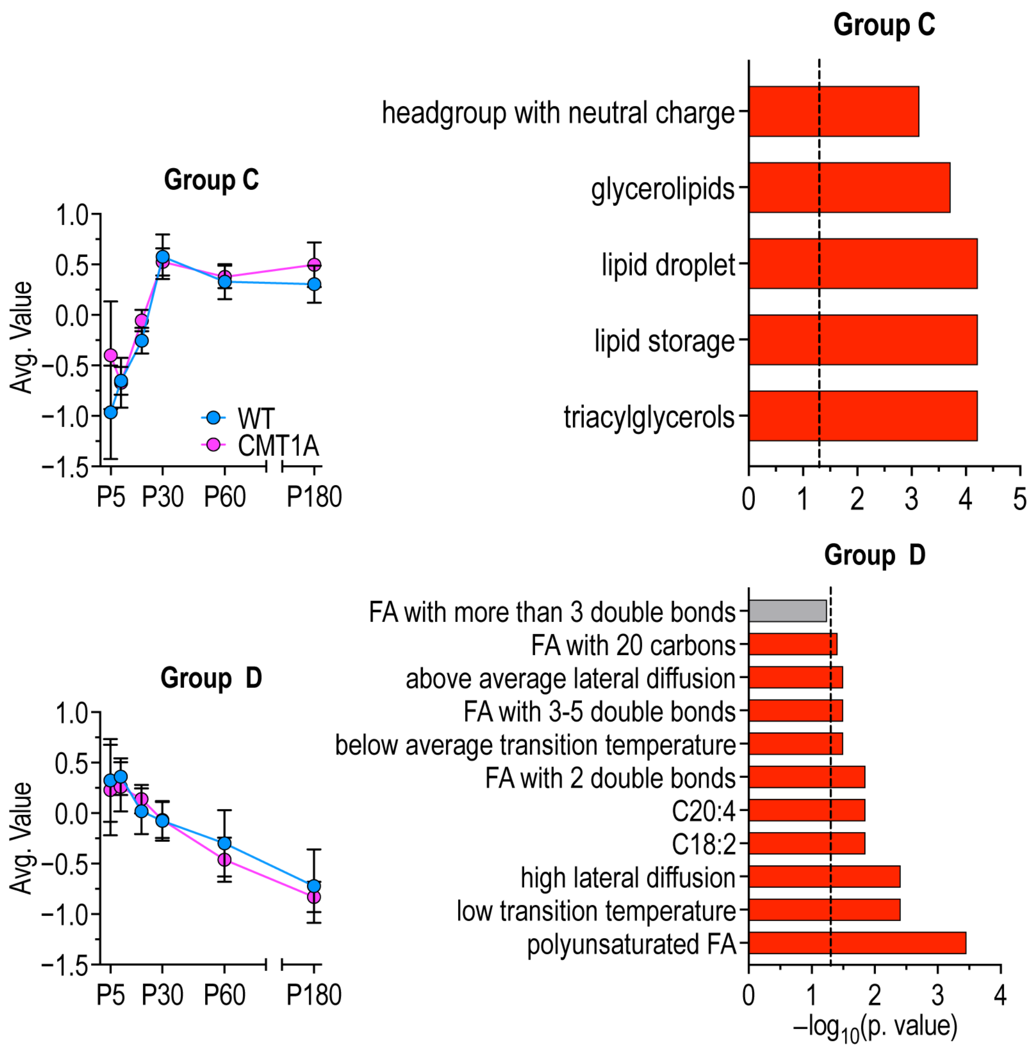

Supplementary Figure S3. Lipidomics Data; enrichment analysis. On the left, average value of lipids in group C (top) and D (bottom); on the right, results of enrichment analysis performed using LION software on lipids in group C (top) and D (bottom). The dotted line marks *p* value significance threshold (0.05).

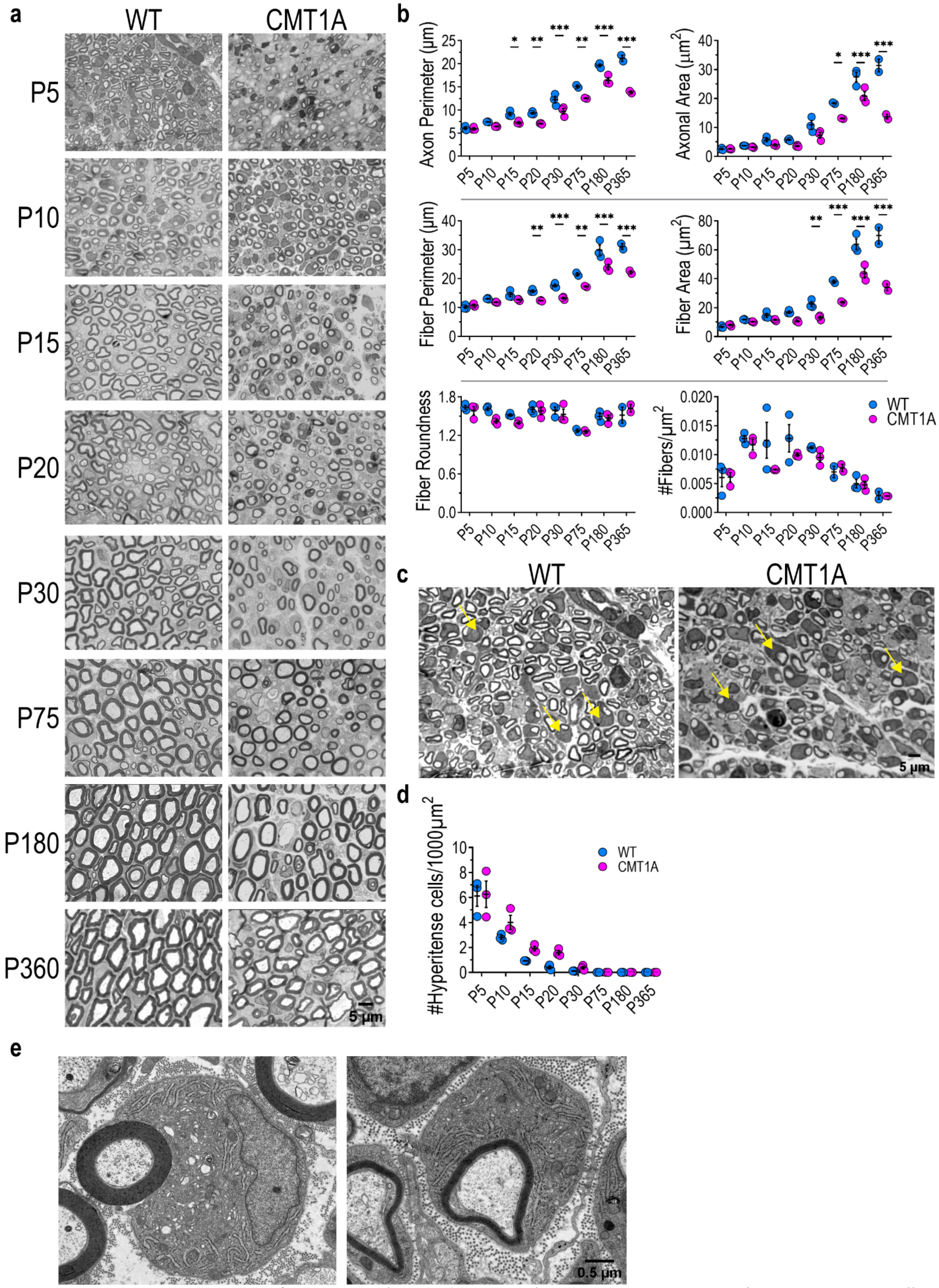

Supplementary Figure S4. Morphometric Parameters and Hyperintense cells. (**a**) Morphometric parameters from WT and CMT1A sciatic nerve sections. (**b**) Micrographs of cross-sections of P5 WT and CMT1A sciatic nerves stained with toluidine blue; yellow arrows indicate hyperintense cells. (**c**) Hyperintense cell density in WT and CMT1A sciatic nerves at different time points. n = 3 rats for each genotype and each time point. At P5, at least 1000 myelinated fibers were evaluated for each rat; at later time points, at least 3000 fibers were evaluated for each rat. (**d**) Transmission electron

microscopy micrographs of representative hyperintense cells. Scale bar: 0.5 µm. Values are presented as mean ± SEM. * $p < 0.05$, ** $p < 0.01$, *** $p < 0.001$. p values were calculated using Kruskal–Wallis test followed by Sidak's multiple comparisons tests between the two genotypes for each time point.

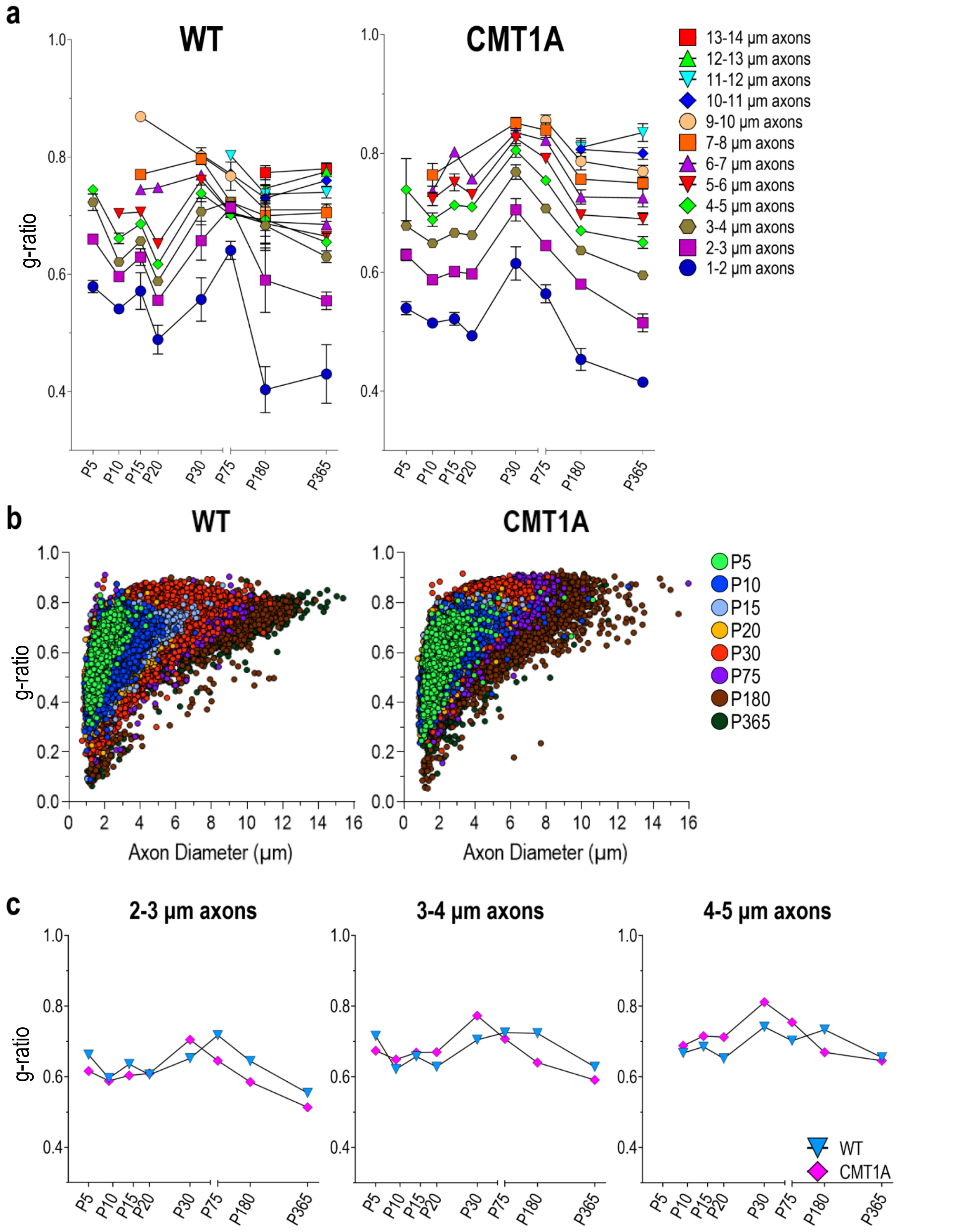

Supplementary Figure S5. Analysis of g-ratio evolution during development (**a**) Developmental trajectories of g-ratio in WT (left) and CMT1A (right) rat sciatic nerves from early postnatal days to adulthood, measured for each axon class. Fibers were classified based on axon diameter. (**b**)

Scatterplots showing the g-ratio plotted against the axon diameter of WT and CMT1A sciatic nerve fibers at different time points. (**c**) Same data as in **a**, but WT and CMT1A data of the same axonal class are plotted together. Values are presented as mean ± SEM; for some values, error bars are not visible because they are covered by the symbol. *** $p < 0.001$. n = 3000 myelinated fibers from 3 different animals per genotype were evaluated at P5, 9000 myelinated fibers from 3 different animals per genotype were evaluated at later time points. *p* values were calculated as in Figure 3c.

Supplementary Materials and Methods

Quantitative fiber morphology

Fiber Roundness was defined as $\pi \times$ diameter/perimeter. This parameter can be used as a parameter of tissue integrity since it is sensitive to physiological strains acting on myelinated fibers during development; for the same reason, it is a useful criterion to exclude from the analysis fibers displaying artifacts due to tissue processing.

Hyperintense cells

"Hyperintense" cells were counted at each time point for the two genotypes. For transmission electron microscopy analysis, 70 nm ultra-thin sections were cut and stained with 1% uranyl acetate and lead citrate solution. Images were collected with a Jeol JEM 1011 (Jeol, Japan) electron microscope, operating with a maximum acceleration voltage of 100 kV, and recorded with a 2 Mp charge-coupled device camera (Gatan Orius SC100).

Data visualization

Network visualization of lipid metabolism was generated using Cytoscape (v. 3.10.1) (https://cytoscape.org/).
